# The Rhizobacterium *Pseudomonas alcaligenes* AVO110 Induces the Expression of Biofilm-Related Genes in Response to *Rosellinia necatrix* Exudates

**DOI:** 10.3390/microorganisms9071388

**Published:** 2021-06-25

**Authors:** Adrián Pintado, Isabel Pérez-Martínez, Isabel M. Aragón, José Antonio Gutiérrez-Barranquero, Antonio de Vicente, Francisco M. Cazorla, Cayo Ramos

**Affiliations:** 1 Área de Genética, Facultad de Ciencias, Campus Teatinos, Universidad de Málaga, E-29010 Málaga, Spain; apintado@uma.es (A.P.); isaperezmart@gmail.com (I.P.-M.); isabel.aragon31@gmail.com (I.M.A.); 2 Departamento de Microbiología y Protección de Cultivos, Instituto de Hortofruticultura Subtropical y Mediterránea «La Mayora», Extensión Campus de Teatinos, Universidad de Málaga-Consejo Superior de Investigaciones Científicas (IHSM-UMA-CSIC), E-29010 Málaga, Spain; jagutierrez@uma.es (J.A.G.-B.); adevicente@uma.es (A.d.V.); 3 Departamento de Microbiología, Campus Teatinos, Universidad de Málaga, E-29010 Málaga, Spain

**Keywords:** *Pseudomonas alcaligenes*, mycophagy, *Rosellinia necatrix*, fungal exudates, c-di-GMP, biofilm formation

## Abstract

The rhizobacterium *Pseudomonas alcaligenes* AVO110 exhibits antagonism toward the phytopathogenic fungus *Rosellinia necatrix*. This strain efficiently colonizes *R. necatrix* hyphae and is able to feed on their exudates. Here, we report the complete genome sequence of *P. alcaligenes* AVO110. The phylogeny of all available *P. alcaligenes* genomes separates environmental isolates, including AVO110, from those obtained from infected human blood and oyster tissues, which cluster together with *Pseudomonas otitidis*. Core and pan-genome analyses showed that *P. alcaligenes* strains encode highly heterogenic gene pools, with the AVO110 genome encoding the largest and most exclusive variable region (~1.6 Mb, 1795 genes). The AVO110 singletons include a wide repertoire of genes related to biofilm formation, several of which are transcriptionally modulated by *R. necatrix* exudates. One of these genes (*cmpA*) encodes a GGDEF/EAL domain protein specific to *Pseudomonas* spp. strains isolated primarily from the rhizosphere of diverse plants, but also from soil and water samples. We also show that CmpA has a role in biofilm formation and that the integrity of its EAL domain is involved in this function. This study contributes to a better understanding of the niche-specific adaptations and lifestyles of *P. alcaligenes*, including the mycophagous behavior of strain AVO110.

## 1. Introduction

In recent years, multitrophic interactions have gained the attention of many research groups because of their importance in plant disease development and the assembly of root-associated microbiomes [1,2,3,4,5]. These interactions modulate the colonization and establishment of microorganisms in different environments and ecological niches [6], including the plant rhizosphere [7]. The rhizosphere is considered to be the portion of soil intimately associated with roots and has been described as one of the most diverse and complex soil environments on the Earth [7,8]. The rhizosphere is strongly influenced by plant root exudates [9], and root exudates are considered to be the major nutrient source for microorganisms in this environment, influencing the assembly of specific fungal and bacterial communities [7,10,11]. Additionally, certain microorganisms can obtain nutrients from living fungi, a process that has been defined as mycophagy [12]. In particular, bacterial mycophagy was first demonstrated in a soil bacterium of the *Collimonas* genus [13], which became the model bacterium for disentangling the mechanisms governing this feature [14,15,16,17,18,19,20]. Although only a few soil bacteria present this feeding ability [12], a more recent study reported that a substantial number of rhizobacteria are able to feed on fungal exudates [21].

*Pseudomonas alcaligenes* (formerly *Pseudomonas pseudoalcaligenes*) AVO110, an efficient avocado root tip colonizer, exhibits in vitro and in planta antagonistic activity toward the soil-borne phytopathogenic fungus *Rosellinia necatrix* [22]. *P. alcaligenes* AVO110 is able to feed on *R. necatrix* exudates and profusely colonize its hyphae without lysing host cells [1], providing evidence for the mycophagous ability of this bacterium as an extracellular biotroph. In addition, *P. alcaligenes* AVO110 forms biofilms on *R. necatrix* mycelia and avocado root surfaces [1,22]. Prior to biofilm development on plant roots, competitive rhizosphere colonization takes place, a process involving bacterial motility [23,24]. However, only a few studies have addressed the identification of bacterial genes involved in interactions with fungal phytopathogens [16,20,25,26,27].

In this sense, a recent study identified several molecular mechanisms linked to the mycophagous behavior of *P. alcaligenes* AVO110 toward *R. necatrix* exudates [28]. Using signature-tagged mutagenesis (STM) [29], these authors identified a collection of AVO110 genes required for growth and survival in *R. necatrix* exudates, several of which are biofilm related. For example, the *algQ* gene, which encodes a regulator of alginate production, was identified as being necessary for efficient colonization of the avocado rhizosphere. Production of the exopolysaccharide (EPS) alginate has been shown to influence biofilm architecture in *Pseudomonas aeruginosa* [30]. In addition, another gene identified in that study encoded a GGDEF/EAL domain protein, named CmpA, from cyclic-di-GMP (c-di-GMP)-metabolizing protein. This gene was shown to be involved in the colonization of both the avocado rhizosphere and fungal hyphae. Among other genes, transcription of *cmpA* has been shown to be induced in response to *R. necatrix* exudates [28]. GGDEF and EAL (or HY-GYP) protein domains are involved in the turnover of c-di-GMP, a universal intracellular second messenger mediating phenotypic changes related to the transition between sessility and motility, such as the production of extracellular matrix components (EPSs and proteins), biofilm formation, and bacterial motility. Cellular c-di-GMP levels have been related to the regulatory network that controls root colonization in plant-associated rhizobacteria [31,32] and virulence traits in bacterial phytopathogens [33,34,35,36]. The GGGDEF and EAL domains are encoded in enzymes responsible for the synthesis (diguanylate cyclases, DGCs) and breakdown (phosphodiesterases, PDEs) of c-di-GMP, respectively [37,38,39].

In this study, we obtained the complete genome sequence of *P. alcaligenes* AVO110 and performed a comparative analysis with the genomes of related pseudomonads of the *P. aeruginosa* and *P. oleovorans* groups. The genome of *P. alcaligenes* AVO110 encodes a variable region of approximately 1.6 Mb (1795 genes), which is absent in all other sequenced *P. alcaligenes* strains. Remarkably, this exclusive region encodes a broad collection of genes related to bacterial lifestyle transitions, such as flagellar motility, chemotaxis, adhesion, biofilm formation, and colonization of host surfaces, 25 of which encode GDEEF and/or EAL domain proteins. Annotation of the *P. alcaligenes* AVO110 genome, followed by transcriptional analyses, allowed us to identify several genes transcriptionally modulated by *R. necatrix* exudates, some of which are encoded in its variable genomic region and are related to cell adhesion and biofilm formation. We also showed that CmpA had a role in biofilm formation and that the integrity of its EAL domain was required for this function.

## 2. Materials and Methods

### 2.1. Bacterial Strains, Media, and Growth Conditions

The bacterial strains used in this study are listed in Table 1. *Pseudomonas* and *Escherichia coli* strains were grown in lysogeny broth (LB) medium [40] or super optimal broth (SOB) [41] at 28 and 37 °C, respectively. BM minimal medium [42] was used for growth of *R. necatrix* Rn400 mycelia to obtain fungal exudate-containing medium (BM-RE, pH 7.1), as previously described [1,28]. When necessary, solid and liquid media were supplemented with antibiotics as follows: for *E. coli*, ampicillin (Ap) 100 µg mL^−1^, kanamycin (Km) 50 µg mL^−1^, and gentamicin (Gm) 50 µg mL^−1^ final concentration; for *P. alcaligenes* AVO110, Ap 300 µg mL^−1^, Km 25 µg mL^−1^, and Gm 10 µg mL^−1^; and for *P. putida* KT2440, Km 50 µg mL^−1^ and Gm 50 µg mL^−1^.

### 2.2. Construction of Bacterial Strains and Plasmids

All recombinant DNA techniques, including restriction digestion, agarose gel electrophoresis, purification of DNA fragments, and ligation with T4 DNA ligase, were performed as previously described [47]. Plasmids were purified using the GenEluteTM Plasmid Miniprep kit (Sigma-Aldrich, Burlington, VT, USA) and, when necessary, sequenced by STAB VIDA Lda. (Caparica, Portugal). DNA amplifications were performed by polymerase chain reaction (PCR) with a standard enzyme (GoTaq^®^ Flexi DNA Polymerase, Promega, Madison, WI, USA) or, for cloning, with Expand High Fidelity polymerase (Roche Applied Science, Mannheim, Germany).

The plasmids and oligonucleotides used for plasmid construction and mutagenesis are listed in Table 1 and Supplementary Materials Table S1, respectively. Construction of the *P. alcaligenes* AVO110 Δ*cmpA* mutant was performed by marker exchange mutagenesis as follows: First, DNA fragments of approximately 1 kb corresponding to the 5′ and 3′ flanking regions of *cmpA* were amplified by PCR using appropriate primers and *P. alcaligenes* AVO110 genomic DNA as a template. The PCR products were joined by the BamHI restriction sites included in the primer sequence, and after cloning the resulting fragment into pBluescript SK II using EcoRI/NotI sites, the fragment was sequenced to verify the absence of mutations. Then, the plasmid was labeled with the *nptII* Km resistance gene obtained from pGEM-T-KmFRT-BamHI, yielding pBSKII-Δ*cmpA*-Km. For marker exchange mutagenesis, plasmid pBSKII-Δ*cmpA*-Km was transformed by electroporation into AVO110, as previously described [48]. Transformants were selected on LB medium containing Km, and replica plates of the resulting colonies were generated on LB-Ap plates to determine whether each transconjugant underwent plasmid integration (Ap-resistant, Ap^R^) or allelic exchange (Ap-sensitive, Ap^S^). Southern blot analysis was used to confirm that the allelic exchange occurred at a single site and at the correct position within the genome.

To overexpress the *P. alcaligenes* AVO110 *cmpA* gene, a 3.6 kb PCR fragment encoding the complete *cmpA* ORF and its ribosome binding site was cloned into pGEM-T Easy and sequenced. The resulting plasmid (pGEM-T-*cmpA*) was digested with EcoRI and SacI, and the EcoRI/SacI fragment encoding *cmpA* was subcloned into the broad host range vector pAMEX, yielding pAMEX-*cmpA*. Site-directed mutagenesis of the GGDEF (pAMEX-*cmpA*-GGAAF) and EAL (pAMEX-*cmpA*-AAL) sites of *cmpA* was performed on pAMEX-*cmpA* using the QuickChange II Site-Directed Mutagenesis kit (Stratagene, San Diego, CA, USA), following the supplier’s instructions.

### 2.3. Complete Sequencing of the P. alcaligenes AVO110 Genome

*P. alcaligenes* AVO110 was grown overnight in LB medium and genomic DNA was extracted using a JetFlex genomic DNA purification kit (Genomed GmbH, Löhne, Germany). The sample was further purified by extraction with phenol–chloroform. Genome sequencing and assembly was performed at BGI Tech Solutions Co., Ltd. (Hong Kong). A library of randomly sheared DNA fragments (0.5–2.0 kb) was subjected to Illumina GA II (Solexa) sequencing. Reads (coverage 100×) were qualitatively assessed before assembling with SOAPdenovo [49]. Primer walking and PCR amplification were used to fill the remaining gaps and solve misassembled regions. The genome (accession number LZEU00000000) was automatically annotated upon submission to GenBank at the National Center for Biotechnology Information (NCBI).

### 2.4. Bioinformatics Methods

Core and pan-genome analyses were performed using BPGA v1.3 [50] with assemblies downloaded from the NCBI. Orthologous genes were identified using the USEARCH algorithm [51] with a threshold of 0.9 (90% Blastp identity). To determine whether pan-genomes were open or closed, we used the medians of the total number of genes found, and then the curves were fitted to Heaps’ law model [52]. The core and pan-genomes of these assemblies were also estimated using Roary v3.12.0 [53] with ≥90% Blastp identity. Phylogenetic relationships were predicted using the estimated core genomes with ≥90% Blastp. Sequences were aligned using MUSCLE and a tree was built using the maximum likelihood method with 100 bootstraps within MEGA 7 [54]. The tree was rooted using the genome of *P. putida* KT2440 as an outgroup.

Variable genomic regions in *P. alcaligenes* strains were identified with the GView Pangenome Analysis tool [55] using *P. alcaligenes* NEB 858 as the seed and Blastn with *e*-value ≤ 1 × 10^−10^ and ≥90% identity. Singleton coding sequences (CDSs) of *P. alcaligenes* AVO110 were extracted and annotated using Sma3s.v2 software [56].

CmpA homologs were identified by Blastp analysis using the *P. alcaligenes* AVO110 amino acid sequence as a query. Identification of conserved protein domains was performed with the Pfam database (Pfam 34.0, http://pfam.xfam.org/, accessed on 30 March 2021).

### 2.5. RNA Techniques

For the RNA-seq analysis, *P. alcaligenes* AVO110 was grown overnight in LB. The next day, the cells were diluted in 100 mL of LB medium to an OD_600_ of 0.05 and grown to an OD_600_ of 0.5. Cells were washed with NaCl 0.9% 3 times and inoculated in 100 mL of BM-RE medium [1] to start the induction. Two samples of 25 mL each were extracted from this volume, and RNA extraction was carried out for one sample at time zero and the other after 4 h of incubation in BM-RE medium. Total RNA was extracted using TriPure isolation reagent (Roche Applied Science) according to the manufacturer’s instructions, except that the TriPure was preheated to 65 °C and the lysis step was performed at 65 °C. Total RNA was cleaned up using an RNAeasy kit (Qiagen GmbH, Hilden, Germany), as detailed by the manufacturer. RNA concentration was determined spectrophotometrically, and its integrity was assessed by agarose gel electrophoresis. Total RNA was treated with a Turbo DNA-free kit (Applied Biosystems, Foster City, CA, USA), as detailed by the manufacturer’s instructions. rRNA depletion using an Illumina RiboZero RNA removal kit, library construction using an Illumina TrueSeq Stranded Sample Preparation kit, Illumina HiSeq 2500 sequencing, and bioinformatics analysis were performed by ChunLab Inc. (Seoul, Korea). Data of differentially expressed genes, provided as CLT format files, were viewed and visualized using Chunlab’s CLRNAseq software (Seoul, Korea).

For the quantitative real-time PCR (qRT-PCR), the induction of *P. alcaligenes* AVO110 cells in BM-RE medium and RNA extraction and purification were performed, as described above for RNA-seq analysis. RNA was extracted from three 25 mL samples at time zero and after 4 h of incubation in BM-RE medium. DNA-free total RNA was reverse transcribed using the cDNA Reverse Transcription kit (Applied Biosystems, Foster City, CA, USA) and random hexamers. The primer efficiency tests, qRT-PCR, and confirmation of the specificity of amplification reactions were performed, as previously described [57]. The relative transcript abundance was calculated using the ΔΔ cycle-threshold (Ct) method [58]. Target cDNAs from the experimental samples were PCR-amplified using 0.3 μM of each primer (Table S1). Transcriptional data were normalized to the housekeeping gene *rpoD*. The qRT-PCR values are the means from 3 biological replicates with 3 technical replicates ± standard deviation.

### 2.6. Biofilm Assays

Biofilm formation by *P. putida* KT2440 overexpressing the wild-type *cmpA* gene from *P. alcaligenes* AVO110 or its mutated alleles, *cmpA*-GGAAF and *cmpA*-AAL, was carried out as previously described [59,60]. *P. putida* KT2440 was transformed by electroporation [48] with pAMEX (empty vector), pAMEX-*cmpA*, pAMEX-*cmpA*-GGAAF, or pAMEX-*cmpA*-AAL (Table 1). Transformants were grown overnight in LB-Km medium at 25 °C. The cultures were prediluted in LB to an OD_600_ of 0.1 and subjected to six 10-fold serial dilutions. Samples (150 µL) of the diluted suspensions were deposited in the wells of a microtiter dish, and the plates were incubated for 20 h at 25 °C with moderate shaking (150 rpm). Wells were subsequently emptied by inverting the plates onto filter paper and washed 3 times by soaking the plates in water. The plates were dried on filter paper and the attached biofilms were stained for 15 min with 200 μL of 0.1% crystal violet. The wells were emptied and again washed 3 times to remove unbound dye. Finally, the wells were filled with 200 μL of 96% ethanol and the plates were incubated for 20 min at room temperature with vigorous shaking (600 rpm). Biofilm growth was determined from the A_630_ reading using a plate reader (FL600 Fluorescence Microplate Reader; Bio-Tek, Winooski, VT, USA).

## 3. Results

### 3.1. Sequencing and General Features of P. alcaligenes AVO110 Genome

The sequencing of the *P. alcaligenes* AVO110 genome yielded a large 4.95 Mb contig encoding 4474 genes and two additional small contigs of 15.6 and 5.8 kb encoding only 13 and 5 genes, respectively. No plasmid-related genes were found among the annotated products of these two small contigs.

The total sequence length of the genome (4.97 Mb) and its number of coding sequences (4406) are within the range of the other 10 *P. alcaligenes* and two *P. pseudoalcaligenes* genomes currently available at NCBI, two of which correspond to the *P. alcaligenes* type strain deposited in two collections (NCTC 10367 and NBRC 14159). However, the 11 *P. alcaligenes* genomes showed a wide range of sizes ranging from 3.68 Mb (strain Bin_52_1) to 7.03 Mb (strain OT 69). Noticeably, the genomes of *P. alcaligenes* strains isolated from infected human blood (MRY13-0052) or oyster tissues (OT 69) were, on average, approximately 2.5 Mb larger than those of strains isolated from water (six strains from wastewater, swimming pools, and aquatic environment of duckweeds) or rhizosphere soil (strain AVO110). Nevertheless, the total G+C content of the *P. alcaligenes* AVO 110 genome (64.9%) was similar to that of the other 10 *P. alcaligenes* genomes (64.1–66.5%) but differed from the approximately 62% G+C characteristics of *P. pseudoalcaligenes* (Table 2).

### 3.2. Phylogenetic Analysis of P. alcaligenes and P. pseudoalcaligenes Strains

**Figure 1:**
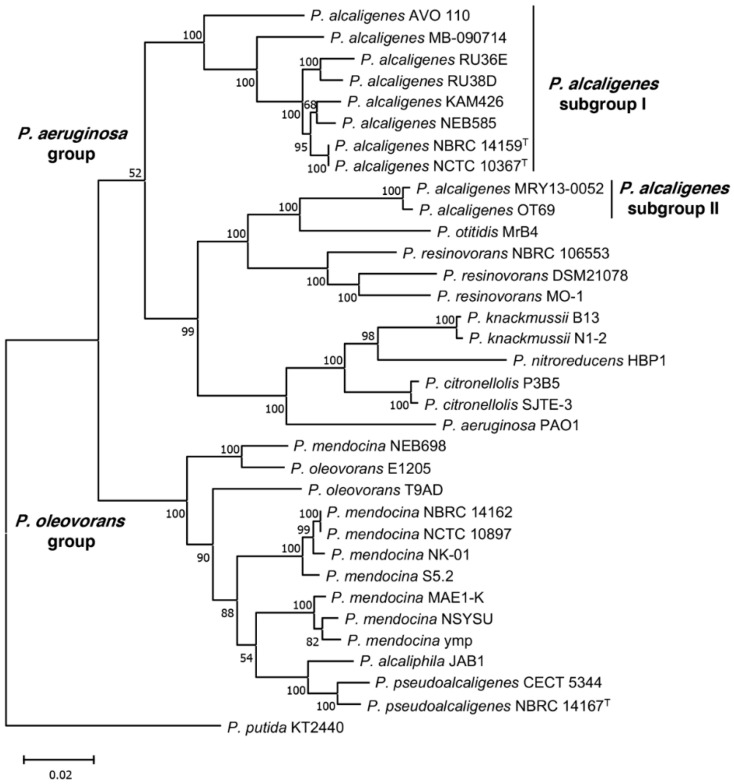
Phylogenetic analysis based on core genome of 34 species belonging to *P. aeruginosa* and *P. oleovorans* groups. The tree was built using MEGA7 [54] and maximum likelihood method with 100 bootstraps. Sequences were downloaded from NCBI and *P. putida* KT2440 was used as an external group. Values in nodes indicate percentages of bootstrap. Superscript ^T^ indicates type strains; *P. alcaligenes* NCTC 10367 and *P. alcaligenes* NBRC 14159 are the same strain deposited in two different collections.

To further study the inclusion of AVO110 in the species *P. alcaligenes*, we analyzed the core genome phylogeny of this strain and 34 other pseudomonads belonging to the *P. aeruginosa* and *P. oleovorans* groups, including, among others, *P. alcaligenes* and *P. pseudoalcaligenes*, respectively [61,62]. The tree was rooted using the genome of *P*. *putida* KT2440 as an outgroup. The genome sequence of *P. alcaligenes* Bin_52_1, which is fragmented into a large number of scaffolds (Table 2), was not included in this analysis or in the comparative genomic analysis described below. The phylogeny showed separation of the strains into two monophyletic branches corresponding to the *P. aeruginosa* and *P. oleovorans* groups. All *P. alcaligenes* strains were included in the *P. aeruginosa* group. However, while *P. alcaligenes* AVO110 grouped in a monophyletic branch with the *P. alcaligenes* strain NBRC 14159 (NCTC 10367) and all other *P. alcaligenes* strains isolated from water samples *(P. alcaligenes* subgroup I), *P. alcaligenes* strains isolated from blood and oyster infections were included in a different sub-branch of the tree (*P. alcaligenes* subgroup II) together with *P. otitidis* and *P. resinovorans* (Table 2). The *P. pseudoalcaligenes* strains analyzed were both included in the *P. oleovorans* group, which also included *P. mendocina* strains and *P. alcaliphila* JAB1 (Figure 1).

**Table 2:** General genome features of *Pseudomonas* strains analyzed in this work.

Strain ^a^	Subgroup ^b^	Genome Size (Mb)	G+C Content (%)	Coding Sequences	Scaffolds (Number)	Coverage	Source of Isolation	Year of Isolation	Accession Number ^c^	Reference or Sequence Source/Year
***Pseudomonas alcaligenes***										
NCTC 10367 ^T^	I	5.02	64.5	4594	2	100×	Swimming pool water	1961	UGUP00000000	[63]
NBRC 14159 ^T^	I	4.82	64.8	4445	122	131×	Swimming pool water	1961	BATI00000000	[63]
NEB 585	I	4.41	65.5	4072	1	382×	Water sample	1989	CP014784	R. D. Morgan/2016
AVO110	I	4.97	64.9	4406	3	100×	Avocado rhizosphere	2005	LZEU00000000	This study
MB-090714	I	4.01	66.5	3815	16	92.7×	Water (lake)	2009	QJRX00000000	M. Batrich/2018
Bin_52_1	I	3.68	64.6	3700	325	978.2×	Water purification facility	2017	SSFO00000000	B.W. Stamps/2018
KAM 426	I	4.68	64.8	4363	1	93×	Wastewater	2020	AP024354	M. Suzuki/2021
RU38D	I	4.25	65.1	3920	10	262×	Water (duckweeds)	NA	FTNW00000000	S. Lebeis /2016
RU36E	I	4.63	64.1	4246	23	221×	Water (duckweeds)	NA	FTMP00000000	S. Lebeis /2016
OT 69	II	7.03	66.0	6379	223	200×	Oyster tissue	2013	ATCP00000000	[64]
MRY13-0052	II	6.88	65.8	6129	237	16.1×	Blood infection	2013	BATO00000000	[65]
***Pseudomonas pseudoalcaligenes***										
NBRC 14167 ^T^	NAP	4.70	62.2	4200	204	131×	Sinus drainage	1982	BDAJ00000000	D. Wibberg/2013
CECT 5344	NAP	4.69	62.3	4082	1	40×	Water (river)	2005	HG916826	A. Hosoyama/2016

^a^ Superscript ^T^ indicates type strains; NCTC 10367 and NBRC 14159 are the same strain deposited in two different collections; ^b^ *P. alcaligenes* subgroup according to the phylogeny (see Figure 1); ^c^ National Centre for Biotechnology Information.

### 3.3. Pan-Genome and Core Genome of P. alcaligenes

To further analyze the genomic diversity of *P. alcaligenes*, we performed core and pan-genome analyses of *P. alcaligenes* subgroups I and II. The eight strains of *P. alcaligenes* subgroup I yielded a hard pan-genome of 11,778 ortholog groups, of which 974 (8%) constitute the hardcore genome, 4176 (36%) are accessory (encoded in 2–7 genomes), and the remaining 6629 (56%) are singletons (Table 3, Figure S1). These results suggest high genomic diversity in this bacterial subgroup. In fact, using Heaps’ law model [52], we estimated a fitting parameter (γ) of 0.50 for *P. alcaligenes* subgroup I, which is well above the critical threshold of γ = 0, distinguishing closed (γ < 0) from open (γ > 0) genomes.

We also investigated the core and pan-genome profiles for the 10 *P. alcaligenes* strains included in subgroups I and II. As expected for higher genomic diversity, the species hard pan-genome (17,284 genes) and hardcore genome (402 genes, 2%) substantially increased and decreased, respectively as compared with the values obtained for subgroup I alone. Furthermore, the number of singletons increased up to 69% (12,004 genes) (Table 3, Figure S1).

Singleton CDSs in *P. alcaligenes* genomes included in subgroup I were identified using the GView Pangenome Analysis tool. The complete sequence of *P. alcaligenes* NEB 858 was used as a seed in this analysis. With the exception of the synonymous strains *P. alcaligenes* NCTC 10367 and NBRC 14159, which share the same variable region, all other genomes encode variable regions (singletons) not shared with any of the other strains.

**Figure 2:**
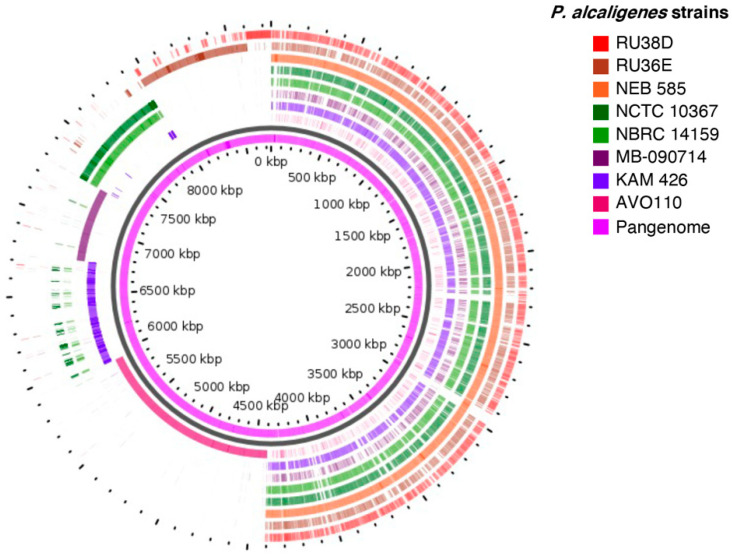
Circular plots of *P. alcaligenes* subgroup I genomes. Genome sequences were subjected to pan-genome analysis using the GView server. Innermost slot (purple) shows constructed pan-genome. White space indicates a region missing in the specified genome.

Remarkably, *P. alcaligenes* AVO 110, the only rhizobacterium from this species that was analyzed, encodes the largest and most exclusive variable region, which covers approximately 1.6 Mb and encodes 1795 CDSs (Figure 2). The clusters of orthologous groups (COG) analysis of the *P. alcaligenes* AVO110 singletons assigned functions to 1483 (82%) of their predicted proteins (Figure S2). The largest COG categories were those related to biosynthetic and metabolic processes, comprising 47% of the proteins assigned to biological processes followed by transport-related proteins (9.0%). Manual curing of COG categories related to cell motility, locomotion, cellular component assembly, and signal transduction (6.8% of the total number of annotated products) revealed that *P. alcaligenes* AVO110 singletons encode 115 genes related to bacterial lifestyle transitions, such as those involved in flagellar motility, chemotaxis, adhesion, biofilm formation, and colonization of host surfaces, 25 of which encode GDEEF and/or EAL domain proteins (Table S2). We also found that 3 of the 21 *P. alcaligenes* AVO110 genes previously identified as required for growth in fungal exudates [28] were singletons, i.e., the GGDEF-EAL domain gene cmpA and two genes potentially involved in the metabolism of fatty acids, fadE and fadD.

### 3.4. Identification of P. alcaligenes AVO110 Genes Transcriptionally Regulated by R. necatrix Exudates

We previously reported the identification of 21 *P. alcaligenes* AVO110 genes required for growth and survival in *R. necatrix* exudates. Transcriptional analysis of five of these genes showed that four of them, including *cmpA*, modulate their expression after transfer to fungal exudates [28]. To further characterize the transcriptional response of this bacterium toward fungal exudates, *P. alcaligenes* AVO110 mid-log phase cells grown in LB were transferred to minimal medium containing *R. necatrix* exudates (BM-RE*).* As a first approach, we performed RNAseq analysis of single biological replicates taken immediately after the transfer (time zero) and after 4 h of incubation in BM-RE. Differentially expressed genes after 4 h in BM-RE relative to time zero (relative fold change (RFC)) were identified. In agreement with our previous results, the transcript levels of the *cmpA* gene increased after transfer to BM-RE (RFC = 3.1). Twelve potentially upregulated genes (RFC = 44.5 to 5.7) and 10 downregulated genes (RFC = 0.08 to 0.5) were selected based on their high and low RFC, respectively. Singleton *P. alcaligenes* AVO110 genes, identified as described above, and genes previously reported to be essential for growth of this bacterium in BM-RE [28], were preferentially selected. The transcript levels of these 22 genes were quantified by qRT-PCR at time zero and after 4 h in BM-RE. Three biological replicates with three technical replicates were used for these assays. Results from qRT-PCR assays confirmed that all 12 upregulated genes, eight of which were *P. alcaligenes* AVO110 singletons, showed increased transcript levels after transfer to the BM-RE medium. However, downregulation of transcription was demonstrated for only 5 of the 10 genes selected, none of which were singletons. The upregulated genes include four genes involved in fatty acid degradation, one of which (*A9179_RS08450*) was a *P. alcaligenes* AVO110 singleton, plus a contiguous regulatory gene, the chemotaxis gene *cheB*, and three genes related to the assembly of the type IVb pili. Downregulated genes encode two glutamine synthetases, a potassium transporter, a metallohydrolase, and an alanine racemase (Table 4).

### 3.5. CmpA, a Pseudomonas spp.-Specific Multidomain Protein

**Figure 3:**
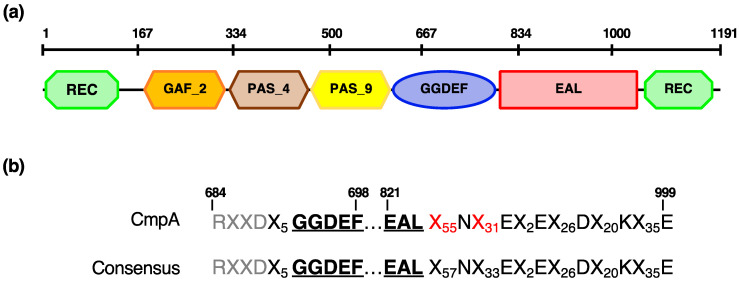
(**a**) Distribution of functional domains present in CmpA protein; (**b**) conservation of GGDEF and EAL domains. Numbers indicate positions of amino acid residues from first methionine. Residues corresponding to RXXD motif, responsible for allosteric control and present in other proteins that contain GGDEF domains, are indicated in gray.

Previously, we reported that the *P. alcaligenes* AVO110 *cmpA* gene encodes a GGDEF/EAL domain protein [28]. Here, we conducted a detailed domain analysis of CmpA using the Pfam database. In addition to the GGDEF and EAL domains, five additional domains were found in this protein: a C-terminal and an N-terminal REC domain, a GAF2 domain, and two tandem PAS domains (PAS4 and PAS9) (Figure 3). All of these domains, which are frequently combined in DGCs and PDEs, are predicted to play a role in small-molecule recognition and in signal transduction via phosphorylation [66,67].

Blastp searches with the sequence of this protein revealed that homologs (>60% identity) exist only within the Pseudomonas genus and include those encoded by 10 strains isolated from nonspecified soil, rhizosphere soil (Pseudomonas indica PIC105, Pseudomonas stutzeri NT0128, and Pseudomonas spp. strains LFM046 and 30_B), hyperthermal compost material (Pseudomonas azotifigens DSM 17556), and water. CmpA homologs conserve the same multidomain structure as that found in *P. alcaligenes* AVO110, with the exception of that in *P. stutzeri* NT0128, which lacks the PAS4 domain. In addition, we found that the PAS9 and GAF2 domains are truncated in the homologs encoded by Pseudomonas sp. ML96 (isolated from lake water) and the soil bacterium Pseudomonas sp. LAM-KW06, respectively (Table 5).

### 3.6. Role of CmpA in Biofilm Formation

Previous studies have demonstrated the key role of c-di-GMP in the transition between sessile and motile lifestyles in different bacterial species [34], including *Pseudomonas* spp. strains isolated from the rhizosphere [31,32]. To decipher the role of CmpA in motility and biofilm formation, a *P. alcaligenes* AVO110 knockout ∆*cmpA* mutant was constructed (Table 1).

**Figure 4:**
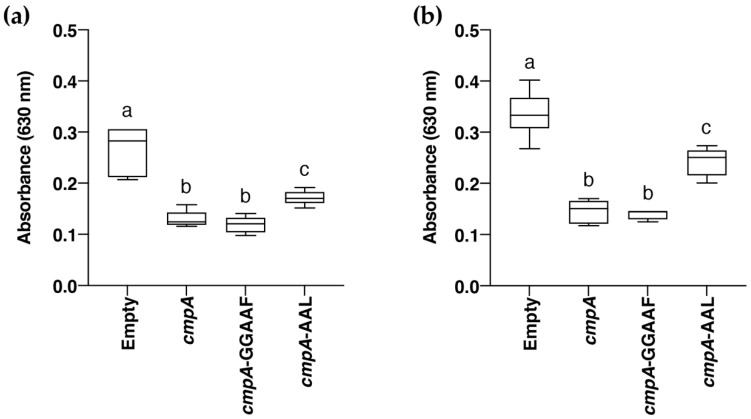
Biofilm formation of *P. putida* KT2440 overexpressing *cmpA* gene from *P. alcaligenes* AVO 110 and its mutated alleles *cmpA*-GGAAF and *cmpA*-AAL. *P. putida* KT2440 LB cultures were subjected to six 10-fold serial dilutions and biofilm assays were conducted on these dilutions. (**a**,**b**) Dilutions of 10^−5^ and 10^−6^, respectively. Biofilm growth was determined from absorbance at 630 nm after crystal violet staining.

However, this mutant showed no differences in swimming, swarming, or biofilm formation in relation to the wild-type strain (data not shown). Thus, we decided to construct a broad host-range plasmid (pAMEX-cmpA) overexpressing the cmpA gene from a constitutive promoter (Table 1). After several attempts, no *P. alcaligenes* AVO110 transformants carrying the plasmid were obtained. Then, we decided to use *Pseudomonas putida* KT2440 as a chassis host for heterologous expression of the cmpA gene. In addition, we performed site-directed mutagenesis of the GGDEF (pAMEX-cmpA-GGAAF) and EAL (pAMEX-cmpA-AAL) motifs of cmpA on plasmid pAMEX-cmpA (Table 1). Independent *P. putida* KT2440 transformants carrying these three plasmids or the empty vector were obtained, and the biofilm formation ability of the strains was assessed using a serial dilution-based method [59]. Overexpression of all three cmpA alleles in *P. putida* KT2440 negatively impacted biofilm formation in relation to the transformant carrying the empty vector, with the sharpest effect being observed using dilutions of 10^−5^ and 10^−6^ as starting inoculum. Expression of wild-type cmpA or cmpA-GGAAF decreased biofilm formation by approximately 2.0-fold (dilution 10^−5^) and by 2.3-fold (dilution 10^−6^) as compared with the wild-type strain transformed with empty vector. However, the ability of *P. putida* KT2440 overexpressing cmpA-AAL to form biofilm was higher than that of the strains expressing the other two alleles (Figure 4). These results reveal a role for CmpA in biofilm formation and determining the involvement of the EAL domain of this protein in this c-di-GMP-related phenotype.

## 4. Discussion

*P. alcaligenes*, a useful bacterium for the biodegradation of toxic polycyclic aromatic hydrocarbons, which is frequently isolated from soil and water [71], has also been known to be a rare opportunistic human pathogen [65]. Recently, several genome sequences of *P. alcaligenes* strains isolated from water or infected human and oyster tissues have been made available (Table 2). However, the *P. alcaligenes* AVO 110 genome obtained in this study represents the first genome of a *P. alcaligenes* strain isolated from the rhizosphere, allowing comparative analysis of niche-specific adaptation and lifestyles in this species, including its mycophagous behavior. Metabolic profiling and partial 16S rRNA gene sequencing initially classified AVO110 as belonging to the species *P. pseudoalcaligenes* [22]. However, results from our phylogenetic analysis (Figure 1) and comparative genome analysis (Table 2 and Table 3, Figure 2) unequivocally assign this strain to the species *P. alcaligenes*. Furthermore, *P. alcaligenes* AVO110 colonies grown on LB medium exhibit a characteristic yellowish-orange pigment, which is not the case with *P. pseudoalcaligenes* strains [72].

In agreement with previous data [61], all sequenced *P. alcaligenes* strains were included in a discrete genomic branch in the core genome phylogenetic tree, which corresponds to the *P. aeruginosa* group. However, separation of *P. alcaligenes* strains into two distinct branches (Figure 1) evidences a different evolutionary history for the strains of subgroup I, isolated from environmental samples (water and rhizosphere), and subgroup II. Subgroup II strains, isolated from infected blood (MRY13-0052) and minced oyster tissue (OT69), clustered together with *P. otitidis* strain MrB4. Although *P. otitidis* was initially isolated from a patient with an ear infection, this species is also widespread in nonclinical environments, as was MrB4, isolated from the near-shore area of Lake Biwa in Japan [73]. In agreement with these results, a recent study showed that a pair-wise comparison of *P. alcaligenes* OT69 against several type-strain genomes indicated *P. otitidis* DSM 17224 as its closest relative, rather than the type *P. alcaligenes* strain NBRC 14159, suggesting that OT69 should be included in a potentially new species [74]. In addition, these authors reported that *P. alcaligenes* OT69 and the other two oyster-associated *Pseudomonas spp.* strains share conserved gene pools, encoding unique metabolic traits that might be recruited via bacteriophage-mediated horizontal gene transfer. Our rarefaction analysis of 10 *P. alcaligenes* genomes belonging to subgroups I and II (Table 3, Figure S1) also showed that both, the large size of the pan-genome and the restricted group of core genes, are likely reflective of the varied lifestyles and niches of members of this species. Thus, the inventory of genes of the *P. alcaligenes* pan-genome will likely continue to increase as new genome sequences become available.

GView identified variable genomic regions in all analyzed *P. alcaligenes* strains of subgroup I, the largest and most exclusive of which is AVO 110 (Figure 2). Functional annotation of the *P. alcaligenes* AVO 110 variable region (singletons) revealed that the largest COG categories were those related to biosynthetic and metabolic processes. One of these genes (*A9179_RS08450*) was found in a cluster of five genes involved in the ß-oxidation of fatty acids, all of which are regulated by *R. necatrix* exudates (Table 4). This cluster also encodes *fadD*, a gene previously identified as essential for the growth and survival of *P. alcaligenes* AVO110 in BM-RE medium [28], suggesting that this bacterium utilizes fatty acid compounds released in *R. necatrix* exudates. In *E. coli*, fatty acid degradation requires the activation of acyl-CoA esters, a process performed by FadD and coupled to transport from the periplasm to the cytosol. Afterwards, degradation of acyl-CoA compounds results in the release of acetyl-CoA, which is oxidized by FadE to enoyl-CoA. In *E. coli, fadD* and *fadE* mutants are unable to grow in oleate and oleic acid, respectively [75]. A *fadE* homolog was also found in the variable region of *P. alcaligenes* AVO 110. Disruption of this gene also disables the ability of this strain to grow in *R. necatrix* exudates [28].

Our COG analysis also revealed more than 100 *P. alcaligenes* AVO 110 singletons related to bacterial lifestyle transitions, such as those involved in flagellar motility, chemotaxis, adhesion, biofilm formation, and colonization of host surfaces (Table S2 and Figure S2). Several studies have reported the relevance of the molecular mechanisms responsible for these phenotypes which underpin the ecological success of bacteria in soil [76], the plant rhizosphere [31,77,78], and fungal surfaces or their exudates [16,26,27,28]. Thus, our analysis of *P. alcaligenes* AVO110 genes transcriptionally modulated by *R. necatrix* exudates particularly focused on a selection of genes potentially relevant to bacterial–fungal interactions and preferentially encoded in the variable genomic region of this strain. Three of the upregulated genes were found to be related to the assembly of type IVb pili (Table 4). Type IVb pili has been shown to be essential for motility, biofilm formation, adherence to eukaryotic cells, colonization, and pathogenesis [79]. In addition, one of the downregulated genes encodes an alanine racemase (Table 4). Alanine racemases catalyze the interconversion of L- and D-alanine, a process essential for peptidoglycan formation and also related to biofilm formation [80].

In a previous study, we reported that the GGDEF/EAL domain-encoding gene *cmpA* was also induced (RFC = 32.27) by *R. necatrix* exudates, and that this gene is involved in the colonization of fungal hyphae and the avocado rhizosphere [28]. Bioinformatics analysis of bacterial genome sequences has led to the identification of GGDEF, EAL, and HY-GYP domains in proteins of all major bacterial phyla. The number of these enzymes greatly varies among bacterial genomes. However, plant-associated bacteria generally encode a high number of proteins with c-di-GMP-related domains. In particular, plant-associated *Pseudomonas spp.* strains encode approximately 30 to 65 proteins that encode one or several of these domains [34]. The *P. alcaligenes* AVO110 genome encodes 58 GGDEF and/or EAL domain-containing proteins, of which 25 (including CmpA) were found in the variable genomic region of this rhizobacterium (Table S2), suggesting that they might have a role in the interaction of this strain with the plant rhizosphere. In fact, CmpA homologs were only found in environmental *Pseudomonas* spp. isolates, most of which were isolated from rhizosphere samples (Table 5).

In addition to the GGDEF and EAL domains, CmpA encodes five additional domains: two PAS, two REC, and a GAF domain (Figure 3). The PAS and GAF domains, commonly found in signaling proteins such as c-di-GMP synthases/hydrolases, are characterized by a ligand-binding pocket interacting with a variety of small-molecule ligands [66]. The REC (receiver) domains are common modules found in a variety of response regulators of bacterial signal transduction systems [67]. Thus, the fusion between a REC domain and various output domains found in CmpA suggests a role for this protein in c-di-GMP-mediated regulation of gene expression or protein–protein interactions in response to environmental challenges. A role for CmpA in c-di-GMP-related phenotypes could not be determined by either construction of a *P. alcaligenes* AVO110 ∆*cmpA* mutant or overexpression of the *cmpA* gene in this strain. Considering that *cmpA* is involved in the colonization of *R. necatrix* hyphae and is overexpressed in response to fungal exudates [28], it may be possible that *cmpA* is not required for motility and biofilm formation in synthetic media or under the conditions used in these assays. Complementation of CmpA by functional overlapping of other GGDEF/EAL/PAS enzymes could also explain these results. In this sense, we found 11 GGDEF/EAL/PAS domain proteins in the genome of *P. alcaligenes* AVO110.

Heterologous expression of *cmpA* in the soil bacterium *P. putida* KT2240 allowed us to establish a role for this gene in biofilm formation. On the one hand, the serial dilution-based method used for this purpose [59] successfully differentiated biofilm formation-defective mutants of *P. putida* KT2440 from the wild-type strain [81]. On the other hand, unlike *P. alcaligenes* AVO110, this strain stability maintains pBBR-MCS derivatives [48], such as pAMEX-*cmpA* (Table 1). In addition, *P. putida* KT2440 does not encode a *cmpA* homolog and contains a sole GGDEF/EAL response regulator [31], a useful characteristic to avoid functional overlapping with similar c-di-GMP signaling circuits. Although a negative impact on biofilm formation was observed by overexpression of wild-type *cmpA* and its mutant alleles *cmpA*-GGAAF and *cmpA*-AAL, the observed decrease was higher for the transformants overexpressing *cmpA* and *cmpA*-GGAAF. Considering that a decrease in biofilm formation usually correlates with a decrease in global c-di-GMP levels in *P. putida* KT2440 and other bacteria [31,34], these results suggests that CmpA exhibits PDE activity, and that the integrity of its EAL motif is involved in this activity. This hypothesis also correlates with the higher ability to colonize the avocado rhizosphere and *R. necatrix* hyphae of a *P. alcaligenes* AVO110 *cmpA* mutant generated by transposon mutagenesis as compared with the wild-type strain [28]. Inactivation of the EAL domain of other GGDEF/EAL/PAS domain-containing proteins also produces an increase in biofilm formation in relation to their wild-type alleles. This is the case with PdeB, a PDE from *Shewanella oneidensis* MR1, a strain that forms biofilms on mineral surfaces [82].

In summary, the sequencing and annotation of the mycophagous rhizobacterium *P. alcaligenes* AVO110 allowed us to identify a variable region in the genome of this strain not shared with other sequenced *P. alcaligenes* strains isolated from water. Our transcriptomics analysis showed that several genes encoded in this region and related to bacterial lifestyle transitions are induced under the influence of exudates from the phytopathogenic fungus *R. necatrix*. We also show that CmpA, a GGDEF/EAL/PAS domain-containing protein specific to *Pseudomonas* spp. strains isolated from environmental samples, has a role in biofilm formation, and the integrity of its EAL domain is involved in this function. Further studies are necessary to demonstrate the PDE activity of this enzyme and its role in c-di-GMP signaling in response to environmental challenges.

## Figures and Tables

**Table 1:** Bacterial strains and plasmids used in this study.

Strain/Plasmid	Relevant Characteristics	Reference or Source
***Pseudomonas alcaligenes***		
AVO110	Wild-type (Nf^R^)	[1,22]
AVO110 ∆*cmpA*	*cmpA* deletion mutant (Nf^R^ Km^R^)	This work
***Pseudomonas putida***		
KT2440	Wild-type (Nf^R^)	[43]
***Escherichia coli***		
DH5α	F^−^, ϕ80d*lac*Z*Δ*M15, *Δ*(*lacZYA*-*argF*)U169, *deoR*, *recA1* *endA1*, *hsdR17*(r_K_^−^ m_K_^+^), *phoA*, *supE44*, λ^−^, *thi-1*, *gyrA96*, *relA1*	[41]
GM2929	F^−^, *ara-14, leuB6, thi-1, tonA31, lacY1, tsx-78, galK2, galT22, glnV44, hisG4, rpsL136, xyl-5, mtl-1, dam13::Tn9, dcm-6, mcrB1, hsdR2, mcrA, recF143* (Sp^R^ Cm^R^)	[44]
**Plasmids**		
pGEM-T Easy vector	Cloning vector, *orif1(-)* and *lacZ* (Ap^R^)	Promega, Madison, WI, USA
pBluescript SK II	Cloning vector, *orif1(-), oripUC,* P*lac, lacZ’* (Ap^R^)	Agilent Technologies, USA
pAMEX	Expression vector, P*nptII* (Km^R^)	[45]
pGEM-T-KmFRT-*Bam*HI	Contains *nptII* gene from pKD4 (Ap^R^ Km^R^)	[46]
pGEM-T-*cmpA*	Contains *P. alcaligenes* AVO110 *cmpA* ORF and its ribosomal binding site	This work
pBSKII-Δ*cmpA*-Km	pBluescript SK II derivative used for marker exchange mutagenesis of *P. alcaligenes* AVO110 *cmpA* gene (Ap^R^ Km^R^)	This work
pAMEX-*cmpA*	pAMEX derivative carrying wild-type *cmpA* gene from *P. alcaligenes* AVO110 (Km^R^)	This work
pAMEX-*cmpA*-GGAAF	pAMEX-*cmpA* derivative carrying mutated *cmpA*-GGAAF allele (Km^R^)	This work
pAMEX-*cmpA*-AAL	pAMEX-*cmpA* derivative carrying mutated *cmpA*-AAL allele (Km^R^)	This work

Nf, nitrofurantoin; Km, kanamycin; Ap, ampicillin.

**Table 3:** Comparison of core and pan-genomes of *Pseudomonas alcaligenes* and *Pseudomonas pseudoalcaligenes* strains.

Species	Subgroup ^a^	Strains	Hard Core Genome ^b^	Soft Core Genome ^b^	Hard Pan-Genome ^b^	Soft Pan- Genome ^b^	Heaps’ Law (Gamma)
*P. alcaligenes*	I	8	974	984	11,778	11,553	0.5030
	II	2	5503	6319	6890	6319	NAP
	I + II	10	402	405	17,284	17,064	0.5817
*P. pseudoalcaligenes*	NAP	2	3301	4009	4872	4009	NAP

^a^*P. alcaligenes* subgroups I and II (Figure 1, Table 2); ^b^ hard- and softcore genomes, genes present in 100 and 95% of genomes, respectively; NAP, not applicable.

**Table 4:** Expression (qRT-PCR) of selected genes in *P. alcaligenes* AVO110 after transfer to *R. necatrix* exudates-containing medium (BM-RE medium).

Transcription	Accession ^a^ (A9179_No.)	Product	Fold Change ^b^
Upregulated	**RS00870**	Chemotaxis-specific protein-glutamate methyltransferase CheB	149.92 ± 55.77
	RS08440	Long-chain acyl-CoA synthetase/AMP-binding protein FadD	389.19 ± 46.96
	RS08445	Long-chain fatty acid-CoA ligase/iron-containing redox enzyme family protein	373.48 ± 68.63
	**RS08450**	Short-chain dehydrogenase/ SDR family oxidoreductase	317.69 ± 94.41
	RS08455	Tetratricopeptide motif repeat protein	107.12 ± 22.51
	RS08460	DNA-binding response regulator	38.27 ± 10.57
	**RS09465**	Pilus assembly protein PilA (type IVb pili)	50.76 ± 11.58
	**RS09470**	Prepilin peptidase (type IVb pili)	29.27 ± 6.44
	**RS09515**	Flp pilus assembly complex ATPase component TadA (type IVb pili)	39.55 ± 4.21
	RS09580 *	*cmpA* (GGDEF/EAL domain-containing protein)	32.27 ± 14,61
	**RS10675**	DUF411 domain-containing protein	94.81 ± 28.25
	**RS10680**	OprD family outer membrane porin	935.76 ± 253.68
	**RS21175**	Hypothetical protein	192.12 ± 85.68
Downregulated	RS00240	Glutamine synthetase	0.17 ± 0.02
	RS10035	Potassium-transporting ATPase subunit KdpA	0.50 ± 0.04
	RS19970	MBL fold metallohydrolase (hydrolysis of beta-lactam antibiotics)	0.39 ± 0.01
	RS21875	Alanine racemase (peptidoglycan biosynthesis)	0.17 ± 0.02
	RS22500	Glutamine synthetase	0.50 ± 0.06

^a^ Bold accession numbers (National Center for Biotechnology Information (NCBI)) indicate singleton *P. alcaligenes* AVO110 genes (see Figure 2), dotted lines separate gene clusters, * data previously reported [28]; ^b^ relative fold changes (normalized to housekeeping gene *rpoD*) after 4 h in BM-RE relative to time zero. Values are means from three biological replicates with three technical replicates ± standard deviations.

**Table 5:** Protein homologs to CmpA from *Pseudomonas alcaligenes* AVO110 in other bacteria.

Strain	Query Cover	Identity	Accession Number ^a^	Source	Reference or Sequence Source/Year
*Pseudomonas* sp. LFM046	100%	78.34%	WP_044875132	Sugarcane soil	[68]
*Pseudomonas* sp. F(2018)	100%	76.91%	WP_171016323	Alpine spring water	O. Jousson/2018
*Pseudomonas* sp. PDM15	100%	76.24%	WP_192398170	Soil	K. Gowda/2020
*Pseudomonas* sp. 30_B	100%	75.99%	WP_207883400	Rice rhizosphere	V. Venturi/2021
*Pseudomonas* sp. TCU-HL1	99%	75.97%	WP_069081885	Soil	[69]
*Pseudomonas* sp. ML96 ^b^	99%	75.32%	WP_043307322	Lake water	X. Li/2014
*Pseudomonas indica* PIC105	99%	71.52%	WP_084333623	*Olea europaea* rhizosphere	C. Gomez-Lama/2017
*Pseudomonas* sp. LAM-KW06 ^b^	99%	70.94%	WP_172149757	Soil	D. Kong/2020
*Pseudomonas azotifigens* DSM 17556	99%	63.30%	WP_028239826	Hyperthermal compost material	[70]
*Pseudomonas stutzeri* NT0128 ^b^	99%	62.51%	WP_052679321	Wheat rhizosphere	N. Tovi/2015

^a^ National Center for Biotechnology Information (NCBI); ^b^ *Pseudomonas spp*. ML96 and LAM-KW06 CmpA homolog encode truncated PAS9 and GAF2 domains, respectively, CmpA homolog from *P. stutzeri* NT0128 does not encode a PAS4 domain (see Figure 3).

## Data Availability

The datasets presented in this study can be found in online repositories. The names of the repository/repositories and accession number(s) can be found in the paper and Supplementary Materials.

## Supplementary Materials

The following are available online at https://www.mdpi.com/article/10.3390/microorganisms9071388/s1, Figure S1: Core and pan-genome analysis of *P. alcaligenes* strains present in (**a**) group I and (**b**) groups I and II. Boxes indicate changes in number of gene families relative to number of genes added sequentially, median values are denoted with a horizontal black line and standard deviation with vertical bars. Pie chart shows frequency distribution of ortholog groups of genes, Figure S2: Predicted biological functions of genes present in specific region of *P. alcaligenes* AVO110. Specific genes were classified by their predicted biological function using Sma3s_v2 software [56], Table S1: Primers used in this study, Table S2: Relevant genes encoded in exclusive genomic region identified with GView in *P. alcaligenes* AVO110.
