# Supplementary material for: The Rhizobacterium Pseudomonas alcaligenes AVO110 Induces the Expression of Biofilm-Related Genes in Response to Rosellinia necatrix Exudates
Source: Microorganisms. 2021 Jun 25;9(7):1388. doi: 10.3390/microorganisms9071388 (PMC8304167; doi:10.3390/microorganisms9071388)
Supplement: Supplementary file 1 [file microorganisms-09-01388-s001.zip › microorganisms-1253869-SI.pdf]

microorganisms

MDPI

Article

The rhizobacterium *Pseudomonas alcaligenes* AVO110 induces the expression of biofilm-related genes in response to *Rosellinia necatrix* exudates

Adrián Pintado [1,2], Isabel Pérez-Martínez [1,2], Isabel M. Aragón [1,2], José Antonio Gutiérrez-Barranquero [2,3], Antonio de Vicente [2,3], Francisco M. Cazorla [2,3,*] and Cayo Ramos [1,2,*]

[1] Área de Genética, Facultad de Ciencias, Universidad de Málaga, Campus Teatinos, E-29010 Málaga, Spain; apintado@uma.es (A.P.; ORCID 0000-0001-6045-3018); isabel.aragon31@gmail.com (IA); isaperezmart@gmail.com (I.P.-M.); crr@uma.es (C.R.; ORCID 0000-0002-2362-5041)

[2] Departamento de Microbiología y Protección de Cultivos, Instituto de Hortofruticultura Subtropical y Mediterránea «La Mayora», Universidad de Málaga-Consejo Superior de Investigaciones Científicas (IHSM-UMA-CSIC); Extensión Campus de Teatinos, 29010 Málaga, Spain; e-mail@e-mail.com

[3.] Departamento de Microbiología, Universidad de Málaga, Campus Teatinos, E-29010 Málaga, Spain; jagutierrez@uma.es (J.A.G.-B.; ORCID 0000-0003-1810-699X); adevicente@uma.es (A.V.; ORCID 0000-0003-2716-9861); cazorla@uma.es (F.M.C.; ORCID 0000-0003-0798-0964)

* Correspondence: crr@uma.es (C.R.), Tel.: (+34-952131957); cazorla@uma.es (F.M.C.), Tel.:(+34-952137587)

Supplementary Material

Figure S1. Core and pan-genome analysis of *P. alcaligenes* strains present in (**a**) group I and (**b**) groups I and II. Boxes indicate changes in number of gene families relative to number of genes added sequentially; median values denoted with horizontal black line and standard deviation with vertical bars. Pie chart shows frequency distribution of ortholog groups of genes.

Figure S2. Predicted biological functions of genes present in specific region of *P. alcaligenes* AVO110. Specific genes were classified by their predicted biological function using Sma3s_v2 software [56].

Table S1. Primers used in this study.

Table S2. Relevant genes encoded in exclusive genomic region identified with GView in *P. alcaligenes* AVO110.

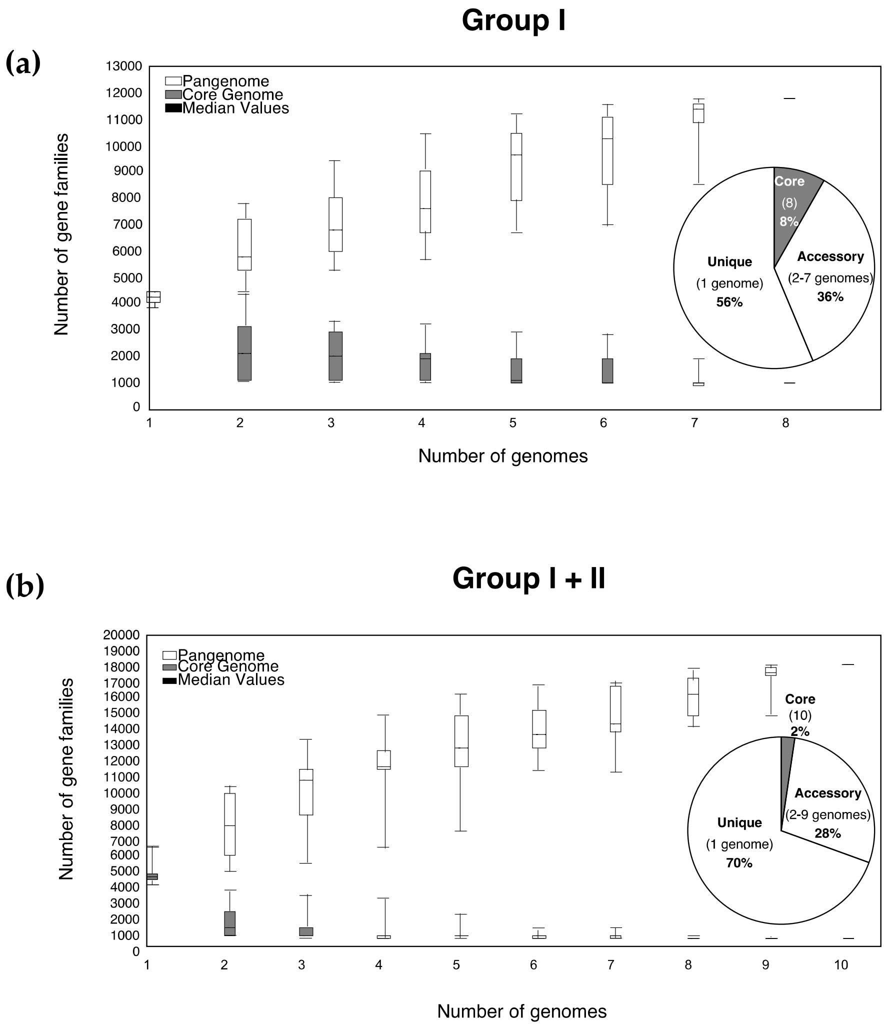

Figure S1. Core and pan-genome analysis of *P. alcaligenes* strains present in (**a**) group I and (**b**) groups I and II. Boxes indicate changes in number of gene families relative to number of genes added sequentially; median values denoted with horizontal black line and standard deviation with vertical bars. Pie chart shows frequency distribution of ortholog groups of genes.

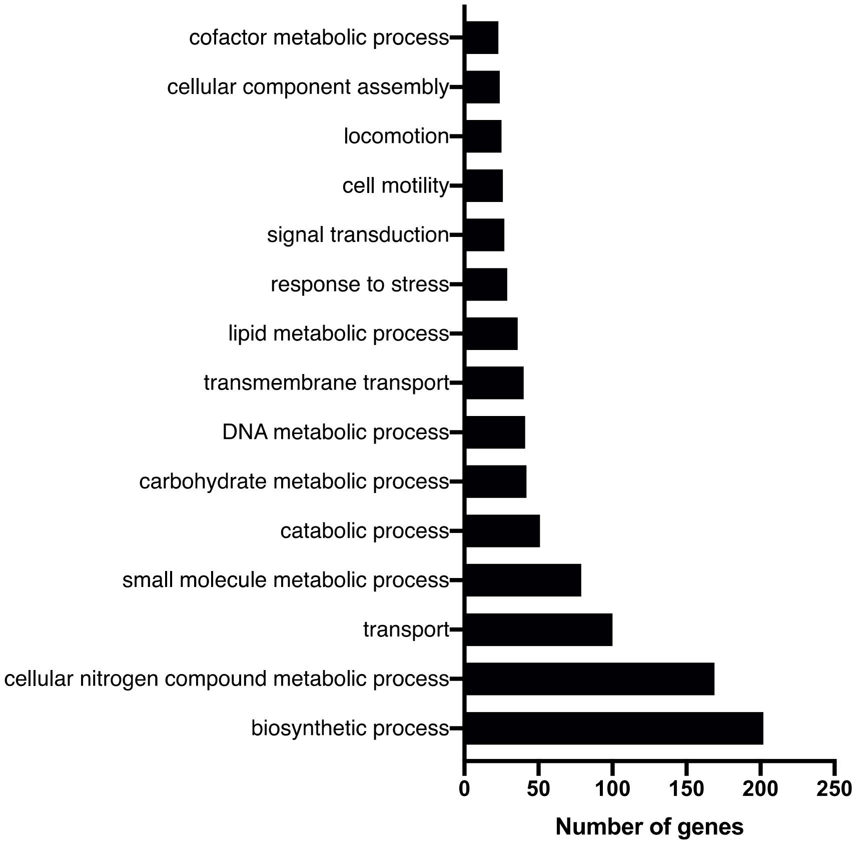

Figure S2. Predicted biological functions of genes present in specific region of *P. alcaligenes* AVO110. Specific genes were classified by their predicted biological function using Sma3s_v2 software [56].

Table S1. Primers used in this study.

Primer name/Use	Sequence 5´ - 3´
**Construction of *P. alcaligenes* AVO110 *ΔcmpA* mutant**	
TAcmpA-F	GAATTCCTGCAGGTGTTGCAGGAC
TAcmpA-R	GGATCCATTCGCATACATGGCTCAGT
TDcmpA-F	GGATCCGCCACGACCATTCATGCG
TDcmpA-R	GCGGCCGCTGTCCACCGCTTCCGGTA
***cmpA* probe**	
cmpA-625-F	ACTGCCTGTTCCACTTCACC
cmpA-1060-R	TGTTCCATGCTCTCGAACAG
**Sequencing of *cmpA* amplicons**	
cmpA-912-F	ATGCTCAATGTGGTGCAGAG
cmpA-2540-R	AGTCGATACCGACGATCCAG
cmpA-544-R	GTTGAGCAGCGAATTGCTGC
cmpA-1088-R	GCAAGCTGTCGAGAATCTTG
***cmpA* construction**	
cmpAEcoRI-F	CCCGAATTCACCTATCTCAACACCCTG
cmpASacI-R	GAGGAGCTCGGCGCATGAATGGTCGT
**Construction of *cmpA*-GGAAF mutant allele**	
GGAAFcmpA-F	TGGGTGGCGCCGCGTTCATCAT
GGAAFcmpA-R	ATGATGAACGCGGCGCCACCCA
**Construction of *cmpA*-AAL mutant allele**	
AALcmpA-F	GGCTTCGCGGCGCTGGTGCGCT
AALcmpA-R	AGCGCACCAGCGCCGCGAAGCC
**qRT-PCR assays of *P. alcaligenes* AVO110 genes**	
19020S-F	GGGTGGTCTGGTGACTATTG
19020S-R	AGCACAGCGTCTTTCAGGC
41690S-F	TCCCGAACAGGTAAACAAGG
41690S-R	CTACCGCCAGATAGGTCTCG
16990S-F	CAGCATGAACAGCGACCAG
16990S-R	CCAGCCCAGGTACAGCTC
19030S-F	CGGTCTGTGTGGATTTCTCA
19030S-F	ATTGAGCGGTTGCCAGAC
17000S-F	AGTTCCACAGGACGACAAGG
17000S-R	CCAACTGCTGGTGACTATGC
16980S-F	TACGTGGCTTTCCTGACCCA
16980S-R	GCACTGCTTGATGGTTGC
21310S-F	GTATTCCCTGTGACGCCTGT
21310S-R	CTGGAGTTTGGTTGGCTCAT
19120S-F	CTGAATGCCGAGAGCCTGGT
19120S-R	ACGTTGAGCATGGTGGTCT

21300S-F	AAGTGGATCTCGCACCTTGA
21300S-R	GCTGCTGGCACATGACCTT
17010S-F	AAGGCTATACGGTGGACTGG
17010S-R	GGGCGGTAAGGATCAGCAC
16970S-F	GGCTCGATTCTCAACGGTTA
16970S-R	TGACGAACTGGTGCTTCTTG
01790S-F	GACGGCATCACCTTCCTG
01790S-R	GCTGTCCTGGAGAAAATCG
00470I-F	ACAAGGTGCCGTTCCTCTAC
00470I-R	GCCAGACCCAGGTAGATCAG
00480I-F	TACAAGAACTGCCGCATCTG
00480I-R	GCGTATTCCAGCAGCACCT
39320I-F	CACCAAGCACTTCAACGAGA
39320I-R	TCGACGAGCACCTTACGC
11670I-F	GATGACCCGTTCCTTCTCC
11670I-R	AGGGCGTCTTCACCGTAGA
00490I-F	ACGGCAACAACCTGATGG
00490I-R	GGTAGGAGTTGGCGTTGG
21360I-F	CCCTCACCACACCTATCAGC
21360I-R	AGCCATTGCTTCGGGTATC
08760I-F	GGCTCGCACTTTCACTTCTT
08760I-R	CTCGACCAGCTCCACCTC
44070I-F	CCCTGTTCAAGAGCAAGTGG
44070I-R	AAAGGTCTGGGTCGTCCTCT
42920I-F	TGCAGGTGTGGCTGAAACTC
42920I-R	GCACGATCTTCTCAACCTTGC
20110I-F	AGCAACAGTGTCGGCAACTT
20110I-R	TCGCATAGTCGAGGAAGGTC
44120I-F	CCGCACCCTCTACTACGC
44120I-R	CTCGGAACTGTCGTTGAGGT
39300I-F	TTCTACGCCGAGGATCTGAG
39300I-R	CGCACGCAGTTCAGGTAGTA
*rpoD*QFwd	GCATCCTCGGTGAATACCAG
*rpoD*QRev	CCATCTCCTTCTTCTTCGTC

Table S2. Relevant genes encoded in exclusive genomic region identified with GView in *P. alcaligenes* AVO110.

Accession number	Description
**c-di-GMP metabolism**	
A9179_RS01010	diguanylate cyclase
A9179_RS02870	diguanylate cyclase
A9179_RS05330	EAL domain-containing protein
A9179_RS05980	EAL domain-containing protein
A9179_RS07085	diguanylate cyclase
A9179_RS07935	diguanylate cyclase
A9179_RS08950	diguanylate cyclase
A9179_RS09580	EAL domain-containing protein (*cmpA*)
A9179_RS10400	diguanylate cyclase
A9179_RS11905	diguanylate cyclase
A9179_RS11915	EAL domain-containing protein
A9179_RS12015	EAL domain-containing protein
A9179_RS12450	EAL domain-containing protein
A9179_RS13275	diguanylate cyclase
A9179_RS13525	diguanylate cyclase
A9179_RS13810	diguanylate cyclase
A9179_RS13845	diguanylate cyclase
A9179_RS14655	EAL domain-containing protein
A9179_RS16485	EAL domain-containing protein
A9179_RS16570	diguanylate cyclase
A9179_RS20705	diguanylate cyclase
A9179_RS21205	EAL domain-containing protein
A9179_RS21270	diguanylate cyclase
A9179_RS22250	EAL domain-containing protein
**Chemotaxis**	
A9179_RS11925	chemotaxis protein
**A9179_RS00870**	**chemotaxis protein CheB**
A9179_RS00875	chemotaxis protein CheD
A9179_RS00885	chemotaxis protein CheW
A9179_RS00895	chemotaxis protein CheW
A9179_RS00910	methyl-accepting chemotaxis protein
A9179_RS02050	methyl-accepting chemotaxis protein
A9179_RS06355	methyl-accepting chemotaxis protein
A9179_RS09555	chemotaxis protein CheW
A9179_RS09560	chemotaxis protein CheR
A9179_RS09565	chemotaxis protein CheW

A9179_RS17360	chemotaxis protein
A9179_RS19375	methyl-accepting chemotaxis protein
A9179_RS20060	methyl-accepting chemotaxis protein
**Flagellar and type IV pili proteins**	
A9179_RS01330	Flp pilus assembly complex ATPase component TadA
A9179_RS02195	PilN domain-containing protein
A9179_RS02205	pilus assembly protein PilP
A9179_RS04850	prepilin-type N-terminal cleavage/methylation domain-containing protein
A9179_RS06045	PilZ domain-containing protein
A9179_RS06055	flagellar type III secretion system protein FlhB
A9179_RS06060	flagellar biosynthetic protein FliR
A9179_RS06065	flagellar type III secretion system protein FliQ
A9179_RS06070	flagellar type III secretion system pore protein FliP
A9179_RS06075	flagellar motor switch protein FliN
A9179_RS06080	FliM/FliN family flagellar motor switch protein
A9179_RS06090	flagellar hook-basal body complex protein FliE
A9179_RS06095	flagellar M-ring protein FliF
A9179_RS06100	flagellar motor switch protein FliG
A9179_RS06105	flagellar assembly protein H
A9179_RS06110	flagellar protein export ATPase FliI
A9179_RS06115	flagellar FliJ family protein
A9179_RS06120	flagellar biosynthesis anti-sigma factor FlgM
A9179_RS06125	flagellar export chaperone FlgN
A9179_RS06130	flagellar filament capping protein FliD
A9179_RS06135	flagellar export chaperone FliS
A9179_RS06145	flagellar hook-length control protein FliK
A9179_RS06150	flagellar basal body-associated FliL family protein
A9179_RS06155	FliA/WhiG family RNA polymerase sigma factor
A9179_RS06160	flagellar motor stator protein MotA
A9179_RS06165	OmpA family protein
A9179_RS06185	flagellar basal body P-ring formation protein FlgA
A9179_RS06190	flagellar basal body rod protein FlgB
A9179_RS06195	flagellar basal body rod protein FlgC
A9179_RS06200	flagellar hook assembly protein FlgD
A9179_RS06205	flagellar basal body protein FlgE
A9179_RS06210	flagellar basal body rod protein FlgF

A9179_RS06215	flagellar basal-body rod protein FlgG
A9179_RS06220	flagellar basal body L-ring protein FlgH
A9179_RS06225	flagellar basal body P-ring protein FlgI
A9179_RS06235	flagellar hook-associated protein FlgK
A9179_RS06240	flagellar hook-associated protein FlgL
A9179_RS09050	flagellar brake protein
A9179_RS09055	flagellar export chaperone FlgN
A9179_RS09060	flagellar biosynthesis anti-sigma factor FlgM
A9179_RS09065	flagellar basal body P-ring formation protein FlgA
A9179_RS09175	flagellar protein FlaG
A9179_RS09180	flagellar filament capping protein FliD
A9179_RS09185	flagellar export chaperone FliS
A9179_RS09190	flagellar export chaperone FliS
A9179_RS09195	flagellar protein FliT
A9179_RS09260	flagellar hook-length control protein FliK
A9179_RS09355	flagellar motor protein MotD
**A9179_RS09465**	**Flp family type IVb pilin**
**A9179_RS09470**	**prepilin peptidase**
A9179_RS09480	Flp pilus assembly protein CpaB
A9179_RS09485	pilus assembly protein N-terminal domain-containing protein
A9179_RS09500	pilus assembly protein
A9179_RS09505	pilus assembly protein
**A9179_RS09515**	**Flp pilus assembly complex ATPase component TadA**
A9179_RS13420	PilZ domain-containing protein
A9179_RS14710	pilus assembly protein PilY
A9179_RS14720	PilW family protein
A9179_RS14730	prepilin-type N-terminal cleavage/methylation domain-containing protein
A9179_RS16440	PilZ domain-containing protein
A9179_RS17450	PilZ domain-containing protein
A9179_RS18550	pilin
A9179_RS18640	PilZ domain-containing protein
A9179_RS18685	prepilin-type N-terminal cleavage/methylation domain-containing protein
A9179_RS18690	pilus assembly protein
A9179_RS18695	pilus assembly protein PilX
A9179_RS18700	prepilin-type N-terminal cleavage/methylation domain-containing protein
A9179_RS20005	flagellin

A9179_RS20010	prepilin-type N-terminal cleavage/methylation domain-containing protein
A9179_RS20015	pilus assembly protein PilY
A9179_RS20025	prepilin-type N-terminal cleavage/methylation domain-containing protein
A9179_RS20130	prepilin-type N-terminal cleavage/methylation domain-containing protein
A9179_RS20135	prepilin-type cleavage/methylation domain-containing protein
A9179_RS20145	prepilin-type N-terminal cleavage/methylation domain-containing protein
A9179_RS20150	prepilin-type N-terminal cleavage/methylation domain-containing protein
A9179_RS20195	PilN domain-containing protein
A9179_RS22575	PilZ domain-containing protein

[a]Bolded accession numbers (NCBI, National Center for Biotechnology Information) indicate genes witch expression was analyzed by qRT-PCR in *R. necatrix* exudates-containing medium (BM-RE medium).

References

56. Casimiro-Soriguer, C.S.; Muñoz-Mérida, A.; Pérez-Pulido, A.J. Sma3s: A universal tool for easy functional annotation of proteomes and transcriptomes. *Proteomics* **2017**, *17*, doi:10.1002/pmic.201700071.
